# Complete genome sequences of *Escherichia coli* phages Huey, Dewey, and Louie

**DOI:** 10.1128/mra.00270-25

**Published:** 2025-06-18

**Authors:** Enea Maffei, Luc Willi, Alexander Harms

**Affiliations:** 1 Biozentrum, University of Basel 27209, Basel, Basel-Stadt, Switzerland; 2 Institute of Food, Nutrition and Health, ETH Zurich 31089 , Zürich, Switzerland; Loyola University Chicago, Chicago, Illinois, USA

**Keywords:** *Escherichia coli*, *Lederbergvirus*, P22, O-antigen

## Abstract

Bacteriophages Huey, Dewey, and Louie are new viruses infecting *Escherichia coli* K-12 that target its O16-type O-antigen as the host receptor. We report the genomes of these phages, which are 38,575, 40,352, and 38,040 bp in size. These viruses belong to the *Lederbergvirus* genus of podoviruses, similar to the well-studied model phage P22, which infects *Salmonella* Typhimurium.

## ANNOUNCEMENT

The recognition of surface-exposed glycans, such as the O-antigen, is a crucial determinant of the host range of phages infecting *Escherichia coli* (1, 2). Phages often display tailspikes that target these glycans with enzymatic activity to direct the virion to the host cell surface for viral genome injection (3, 4). Temperate podoviruses of the *Lederbergvirus* genus, such as P22 (infecting *Salmonella* Typhimurium), Sf6 (infecting *Shigella flexneri*), or HK620 (infecting *E. coli*), have been key models to unravel the biology and molecular mechanisms of tailspike functionality (5–7). Beyond fundamental research, phage tailspikes hold great potential for viral host range engineering with applications in clinics or biotechnology (8). In this study, we report the genomes of three new *Lederbergvirus* phages called Huey, Dewey, and Louie. These phages were isolated from wastewater in 2020 (Huey; sewage treatment plant ARA Basel in Basel, Switzerland at 47°34′53.6″N 7°36′00.9″E) or in 2021 (Dewey and Louie; sewage treatment plant ARA Canius in Lenzerheide, Switzerland at 46°42′33.3″N 9°33′05.7″E). Briefly, wastewater samples were plated on a lawn of *E. coli* K-12 with restored O16-type O-antigen grown in LB agar at 37°C as described previously (2). To identify new isolates that would depend on O-antigen as a host receptor, we also tested their growth on regular *E. coli* K-12 (without restored O16-type O-antigen) and continued to characterize those that formed plaques only on the host with this glycan (2). Three of these isolates formed turbid plaques—indicative of a temperate lifestyle (9)—and were passaged three times for clonal purification before producing high-titer lysates in SM buffer using double-agar overlays (10). Chromosomal DNA was extracted from each 1 mL of these stocks using the Norgen Biotek Phage DNA Isolation Kit. For genome sequencing, libraries were prepared using the protocol of Baym and colleagues (11) and sequenced on the NovaSeq X Plus platform by SeqCenter (Pittsburgh, USA). Adapters were removed using bcl2fastq (version 2.20.0.422; Illumina Inc., San Diego, USA), and trimmed reads (151 bp length) were assembled using the Geneious Assembler implemented in Geneious Prime 2024.0.2. We obtained a circular assembly for each phage with a length of 38,575 bp and a mean coverage of 192× for Huey (from 58,006 reads), 40,352 bp/452× (Dewey; from 128,356 reads), and 38,040 bp/420× (Louie; from 110,804 reads). The genomes were annotated using Pharokka version 1.7.3 and Phold version 0.2.0 with standard settings, after which we set their 5′ ends to the start of the large terminase gene (12, 13). Huey, Dewey, and Louie were identified as *Lederbergvirus* phages using BLAST (version 2.16.0) of the full genomes against the non-redundant nucleotide database of NCBI GenBank (release 262) based on high sequence similarity to previously classified representatives (14, 15). Their phylogenetic relationships to other phages of this genus, including BASEL phage CurroJimenez (Bas106), are unraveled in a parallel manuscript, which also shows the close relationship between tailspikes of these O16-targeting *Lederbergvirus* phages and those of other viruses targeting the same glycan (2). We anticipate that Huey, Dewey, and Louie will not only expand the known diversity of the *Lederbergvirus* genus but may, through their O16-targeting tailspikes, also provide new insights into phage host recognition.

## Data Availability

Whole-genome shotgun projects for Huey, Dewey, and Louie have been deposited under accession numbers PQ850609, PQ850600, and PQ850616 with the genome versions described in this paper as the original versions. The sequencing reads have been deposited in the sequencing read archive (SRA) under accession numbers SRR31909084, SRR31909086, and SRR31909085.
